# Measuring habitat complexity and spatial heterogeneity in ecology

**DOI:** 10.1111/ele.14084

**Published:** 2022-08-17

**Authors:** Lynette H. L. Loke, Ryan A. Chisholm

**Affiliations:** ^1^ School of Natural Sciences, Faculty of Science and Engineering Macquarie University North Ryde New South Wales Australia; ^2^ Department of Biological Sciences National University of Singapore Singapore City Singapore

**Keywords:** box‐counting, complexity–diversity relationships, digital elevation model (DEM), fractal dimension, habitat structure, landscape metrics, photogrammetry, remote sensing, spatial analysis, spatial ecology

## Abstract

Habitat complexity has been considered a key driver of biodiversity and other ecological phenomena for nearly a century. However, there is still no consensus over the definition of complexity or how to measure it. Up‐to‐date and clear guidance on measuring complexity is urgently needed, particularly given the rise of remote sensing and advent of technologies that allow environments to be scanned at unprecedented spatial extents and resolutions. Here we review how complexity is measured in ecology. We provide a framework for metrics of habitat complexity, and for the related concept of spatial heterogeneity. We focus on the two most commonly used complexity metrics in ecology: fractal dimension and rugosity. We discuss the pros and cons of these metrics using practical examples from our own empirical data and from simulations. Fractal dimension is particularly widely used, and we provide a critical examination of it drawing on research from other scientific fields. We also discuss informational metrics of complexity and their potential benefits. We chart a path forward for research on measuring habitat complexity by presenting, as a guide, sets of essential and desirable criteria that a metric of complexity should possess. Lastly, we discuss the applied significance of our review.

## Graphical abstract

Given the rise of remote‐sensing, up‐to‐date and clear guidance on measuring complexity is urgently needed. Here we review how complexity is measured in ecology. We provide a framework for metrics of habitat complexity, and for the related concept of spatial heterogeneity. We demonstrate the pros and cons of various complexity metrics particularly focusing on popular ones; we provide a modern critical examination of fractal dimension and show how and why it is untenable despite its appeal. We discuss informational metrics of complexity, and their potential benefits and chart a path forward for research on measuring complexity in ecology.

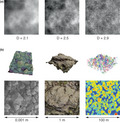

## INTRODUCTION

For nearly a century, ecologists have proposed that habitat complexity promotes biodiversity (Diamond, 1969; Kohn, 1968; MacArthur, 1965; Paine & Vadas, 1969; Pianka, 1969; Rosenzweig & Winakur, 1969) and drives other ecological phenomena (e.g. predator–prey interactions, dispersal patterns; Dice, 1947; Dunlavy, 1935; Gause et al., 1936; Huffaker, 1958; Pimentel et al., 1963; Salt, 1967). Topographically complex habitats, landscapes and regions in both terrestrial and marine systems generally feature disproportionately high biodiversity (Badgley et al., 2017; Kiessling et al., 2010), and therefore have high conservation value (Falk et al., 2006; Ritchie, 2009). In a recent global analysis, Ehbrecht et al. (2021) found that hotspots of enhanced structural complexity coincided with hotspots of greater plant diversity. Even when controlling for available energy (Hurlbert, 2004) and area (Johnson et al., 2003; Loke & Todd, 2016), structurally complex habitats can support greater species richness compared to structurally simple habitats.

**FIGURE 1:**
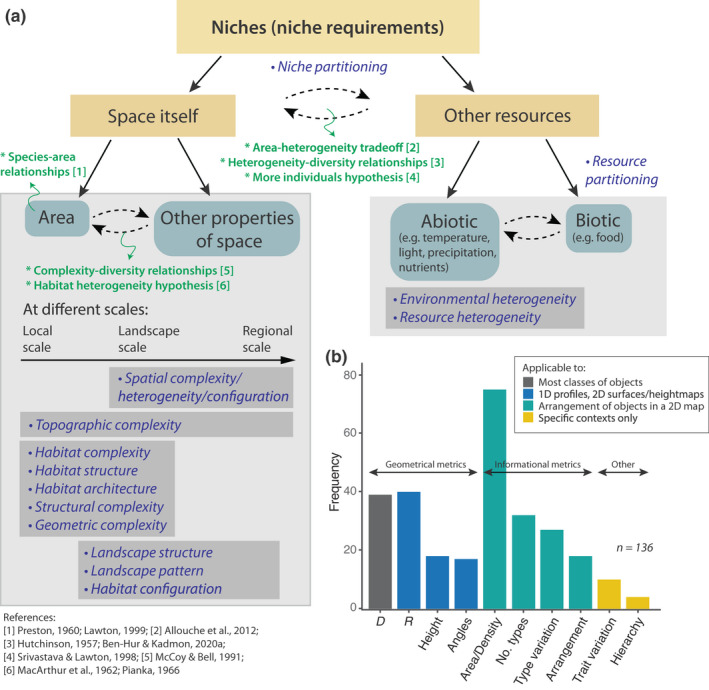
(a) Conceptual diagram of complexity in ecology, including terminology used to describe complexity (blue italicised text) and key relationships on which the effect of complexity has been studied (green text). To describe the complexity of ‘space itself’, various terms are used on different spatial scales (arrow within light grey box at left). To describe the complexity of ‘other resources’, the terms ‘environmental heterogeneity’ and ‘resource heterogeneity’ are broadly used. (b) Metrics of habitat complexity and spatial heterogeneity used in the recent ecological literature, split into four categories. Grey bar: D is fractal dimension (or variations thereof; Section ‘Fractal dimensions’). Blue bars represent other geometric metrics of complexity; R is rugosity (or variations thereof; Section ‘Rugosity’); ‘Height’ is measurements of surface height range including elevation, canopy height, or vertical relief (Section ‘Other geometric metrics of complexity’); ‘Angles’ refers to any measures of angles, slopes, or curvatures (including vector dispersion; Section ‘Other geometric metrics of complexity’). Green bars represent informational metrics of complexity (Section ‘Information‐based metrics of complexity’): ‘Area/Density’ refers to the total area or density of components (e.g. surface area, percent cover, biomass, patch size measurements); ‘No. types’ refers to the number of types of components (e.g. absolute and relative abundance of types or species); ‘Type variation’ refers to variation of components, usually in terms of size (e.g. size range of microhabitats or interstitial spaces); ‘Arrangement’ refers to the spatial configuration of elements (e.g. arrangement of habitat patches in a landscape matrix). Yellow bars: ‘Trait variation’ refers to quantifications of some specific biological or functional group traits (e.g. number of intersections in which a woody plant material contacted an axis of a particular diameter); ‘Hierarchy’ refers to measurements of hierarchical order (e.g. branching order). ‘Area/Density’ is the most frequently used metric here, but it is better thought of as orthogonal to complexity (see main text). Supporting Information S1 describes our bibliographic analysis.

Habitat structure refers to the geometry of the physical habitat; this includes the bare substrate itself (e.g. rock, soil, soft sediments) and the structure provided by the species that characterise that habitat (e.g. macrophytes, trees, oysters, corals; Graham & Nash, 2013; McCoy & Bell, 1991). The geometry of habitat space is important to biodiversity because it directly affects the establishment and persistence of plant and animal communities (McCoy & Bell, 1991; Warfe et al., 2008). For instance, in aquatic habitats, microhabitat features provide refuge from predators and from physical stressors such as turbulence or desiccation (Cardinale et al., 2000; Downes et al., 1998; Gosselin & Chia, 1995; Menge & Lubchenco, 1981). However, there has been little agreement on how exactly habitat structure mediates biodiversity, as measured by patterns including complexity–diversity relationships (see Figure 1a; Hurlbert, 2004). This is partly because complexity is difficult to define, and partly because different ecological processes may be mediated by different aspects of complexity (Ben‐Hur & Kadmon, 2020a; Newman et al., 2019).

Our goal here is to review existing complexity metrics and evaluate their relevance and usefulness to ecology (see Box 1). A complex system is characterised by diverse interacting elements, hierarchical organisation, emergence and related properties (Box 1; Green et al., 2006; Ladyman et al., 2013). Although this definition is not precise, it will guide us in our goal of evaluating complexity metrics, and these metrics will in turn function as operational definitions. There is substantial ambiguity and inconsistency in how complexity in ecology has been measured (Kovalenko et al., 2012; Tews et al., 2004). This ambiguity has been present for more than half a century: MacArthur (1965, p. 515) wrote that ‘some measures of habitat complexity are guessed; to see which, if any, of these measures is responsible for the local bird species diversity’. Used in this way, complexity is presented as an after‐the‐fact explanation for all kinds of ecological patterns. It becomes a catch‐all concept that attempts to compensate for our ignorance about the niche processes that structure ecological communities (Figure 1a). A better understanding of exactly how complexity affects diversity would aid the selection of an appropriate complexity metric in any given application.

BOX 1 **Defining complexity and our objectives**

There are no universally accepted definitions of complexity and complex systems (Gell‐Mann, 1995; Krakauer, 2019), and indeed the appropriate definitions may be domain‐specific (Ladyman et al., 2013). Nevertheless, most definitions of complexity share commonalities in terms of their characterisations of the core features of complex systems: nonlinearity, feedback, disorder, lack of central control, variety of elements and interactions, emergence, and hierarchical organisation (Green et al., 2006; Mitchell, 2009; see framework in Ladyman & Wiesner, 2020). Some of these features are necessary conditions for complexity, while others are merely associated with complexity (see Ladyman et al., 2013). One general definition of complexity, for instance, is that ‘a complex system is an ensemble of many elements which are interacting in a disordered way resulting in robust organization and memory’ (Ladyman et al., 2013, p. 57), where memory is the ‘persistence of internal structure’ (Holland, 1992).

In ecology, a variety of terms are associated with ‘complexity’ and ‘heterogeneity’ (Li & Reynolds, 1995; Tews et al., 2004; see Figure 1a). There is substantial ambiguity among these terms; for example, ‘topographic complexity’ and ‘structural complexity’ describe overlapping but not identical concepts. The variety of terms and ambiguity among them arises partly because of a lack of clarity about which dimensions of complexity are important. One solution is to sidestep the verbal definitional issues and instead develop quantitative metrics that capture aspects of complexity that are believed, based on biological considerations, to be important to organisms. Indeed, of the many complexity metrics in use, each quantifies a subset of the core features of complexity rather than the phenomenon as a whole (Gell‐Mann, 1995; Gell‐Mann & Lloyd, 1996; Ladyman & Wiesner, 2020). In the absence of a strict verbal definition of complexity, these measures of complexity act as operational definitions.

In this Synthesis, we review and evaluate the existing complexity metrics in ecology—we do not attempt to adjudicate on the broader and more contentious issue of the verbal definition of complexity itself. More specifically, our goal is to lay out what dimensions and features of complexity each metric measures and to evaluate whether or not each is useful and relevant to ecology. We present criteria for an ideal metric of complexity (Section ‘Ideal qualities of a metric of complexity’) for use as a guide to identifying and developing metrics that will serve ecology best going into the future, but we do not attempt to establish a universally best metric.

Complexity metrics that have been used in ecology can be broadly classified into three classes: informational, geometric and other (Figure 1b; Gratwicke & Speight, 2005). Informational metrics are broadly applicable across the sciences and measure the amount of information needed to encode and describe an object. Geometric metrics relate more specifically to the physical structure of an object in 2D or 3D space. In ecology, for example, a geometric metric could measure the structural complexity of the surface of a coral reef, whereas an informational metric could measure the spatial organisation of individual corals over the reef. A common informational metric is entropy, which can be influenced by the number of types of elements (e.g. coral species on a reef), variation across types (e.g. in coral sizes) and spatial arrangement of elements (e.g. clustering metrics). Geometric metrics include fractal dimension and rugosity (Warfe et al., 2008). Lacking clear guidance, many ecologists, when attempting to measure complexity, simply choose a metric that is straightforward to measure and characterises what is perceived to be ‘complex’ at some particular scale (Figure 1b; Davenport et al., 1996; McAbendroth et al., 2005).

To further complicate matters, in ecology the term ‘heterogeneity’ is often used interchangeably with ‘complexity’ (Kovalenko et al., 2012; Loke et al., 2015; Tews et al., 2004; Tokeshi & Arakaki, 2012; Figure 1a). In this paper, we treat ‘heterogeneity’ and ‘spatial heterogeneity’ as subsets of ‘complexity’ (see Section ‘Information‐based metrics of complexity’). Another term, ‘environmental heterogeneity’ refers to variation in abiotic conditions across a landscape (e.g. soil moisture; Liu et al., 2018; Srivastava & Lawton, 1998), and has been shown to be an important driver of beta‐diversity (Liu et al., 2018; St. Pierre & Kovalenko, 2014), but we subsume this under ‘spatial heterogeneity’ here (Box 1).

The rapid rise of remote sensing, mapping and photogrammetric technology is enabling an unprecedented increase in the spatial extent and resolution of habitat structural data, and the possibilities for how we might measure complexity are quite different now than they were even ten years ago (Bayley et al., 2019; D'Urban Jackson et al., 2020; Figueira et al., 2015; Friedman et al., 2012; Lawrence et al., 2021). Both 2D digital elevation maps (heightmaps) and 3D vector representations of physical objects facilitate fast computation of complexity metrics. There is a renewed need to understand the pros and cons of various complexity metrics. Ultimately better measurements of complexity can inform our understanding of how complexity interacts with diversity and which aspects of habitat structure (Figure 1a) are important for maintaining biodiversity. In this paper, we review metrics of habitat complexity, in particular focusing on the two most broadly applicable and commonly used metrics in ecology: fractal dimension (D) and rugosity (R). We show why progress towards understanding complexity–diversity relationships is difficult without first resolving obstacles relating to measuring complexity, and outline what qualities a good metric of complexity should possess. We compare the strengths and weaknesses of geometric metrics of complexity with those of informational metrics.

## IDEAL QUALITIES OF A METRIC OF COMPLEXITY

The following is a list of the qualities that an ideal metric of complexity in ecology should possess:

1. It should be broadly applicable to multiple classes of objects (e.g. surface habitats, forests, landscape patterns).
2. Within each class, it should be analytically well defined so as to minimise observer bias and arbitrary context‐dependent classifications. This way, the complexity of different habitats within each class can be compared.
3. It should be practical to measure using current technology (at least at one spatiotemporal scale).
4. It should be decoupled from the effects of area (i.e. there should not be a casual effect of area on the metric).

In addition, there are some desirable but not essential attributes of a complexity metric:

1. It should capture the complexity of aspects of the environment that are, on a theoretical basis, thought to be important to organisms.
2. It should be measurable across multiple spatiotemporal scales.

## METRICS OF COMPLEXITY

### Fractal dimensions

A fractal is a subset of Euclidean space with non‐integer topological dimension. Most well‐known fractals are self‐similar fractals, which means that a magnified version of a small region appears statistically identical to a larger region. Classic examples include Koch's curve and the Sierpinski triangle. A more general class of fractals is self‐affine fractals, that is fractals that scale differently along different dimensions.

Fractal dimension (D) is a measure of the complexity of a fractal object whose behaviour is scale invariant. Loosely speaking, it measures the space‐filling capacity of the object independently of area. Originally, D was used to describe perfectly self‐similar fractals, but it can be adapted to self‐affine fractals (Turcotte, 1997).

**FIGURE 2:**
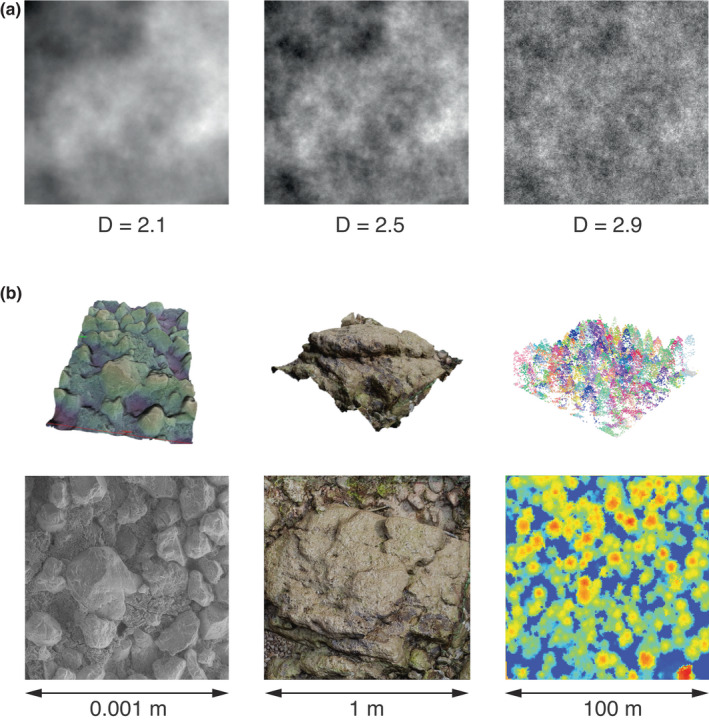
(a) Fractal Brownian surfaces of known fractal dimensions generated by the midpoint displacement algorithm. (b) Examples of fractal‐like empirical surfaces represented in 3D (top row) and 2D (bottom row); from left to right: SEM scan of a 1 mm × 1 mm rock surface, digital elevation model (DEM) of a 1 m × 1 m quadrat on a rocky shore, lidar point cloud of a 100 m × 100 m aerial scan of a forest landscape (image generated using data from the ‘lidR’ package in R; Roussel et al., 2020). Five orders of magnitude in spatial scale are represented by these examples.

Examples of self‐affine fractals are the curves generated by fractal Brownian motion (fBm). Zooming in on a profile of one‐dimensional fBm (e.g. the Weierstrass–Mandelbrot (WM) curves), for instance, does not reveal an object that is statistically self‐similar but instead one that becomes statistically self‐similar only after an appropriate rescaling of one of the axes. Specifically, if a fBm profile with Hurst exponent 𝐻 (0 < 𝐻 < 1) is defined by a function 𝐵_*H*_(𝑡), then

(1) $$B_{H}\left(\mathrm{at}\right)∼{\left|a\right|}^{H}B_{H}\left(t\right),$$

where the tilde (~) means ‘has the probability distribution of’ subject to some normalising constant. The fractal dimension of the curve is D=2−H, because the curve is embedded in two‐dimensional space (e.g. a coastline). A related equation describes the self‐similarity of a two‐dimensional fBm surface, in which case the fractal dimension is D=3−H, because the surface is embedded in three‐dimensional space (e.g. the surface of a mountainous landscape; see also Figure 2).

Objects in nature tend to resemble self‐affine fractals rather than self‐similar fractals because they do not scale similarly in all directions. For example, small‐scale topographical relief may resemble a microscopic version of a mountainous landscape, but only if the vertical axis is scaled differently than the horizontal axes. Indeed, many algorithms used to generate synthetic terrain are based on self‐affine fractals, with the midpoint‐displacement algorithm (based on 2D Brownian motion; Figure 2a) and its variations (e.g. fBms generated by Fourier synthesis or the Voss algorithm; Voss, 1986) being widespread (see Saupe, 1988).

#### Pitfalls of using fractal dimension as a metric of complexity

Since a seminal paper and book by Mandelbrot (1967, 1982) and a classic paper by Sugihara and May (1990) on the application of fractals in ecology, fractal dimension (D) has become a widely used metric of complexity in ecology owing to several convenient and promising features. It is in theory scale invariant (and thus there is no causal effect of area, satisfying criterion (4) in Section ‘Ideal qualities of a metric of complexity’) and as a result it can potentially address problems of scale and hierarchy in ecology. It can also be applied to most geometric objects (satisfying criterion (1) in Section ‘Ideal qualities of a metric of complexity’), and thus allow comparisons of complexity across different systems.

Many studies have attempted to calculate D and use it as a proxy for complexity (and to quantify the ‘spatial heterogeneity’ of landscapes; Milne, 1988) that is then correlated with diversity (e.g. Schindler et al., 2008; see Section ‘Limitations of fractal dimension’) and other ecological variables (e.g. O'Neill et al., 1988; Ritchie, 2009; Ritchie & Olff, 1999). However, while D is well defined in theory, in practice its measurement is fraught with difficulties (see Section ‘Sources of error in estimating fractal dimension of natural objects and patterns’). Furthermore, its relevance to biological organisms is questionable (see Section ‘Limitations of fractal dimension’). The multitude of problems relating to the estimation of D have long been recognised by mathematicians (Dubuc et al., 1989; Huang et al., 1994; Stoyan & Stoyan, 1994) but have generally been ignored by ecologists (Dibble & Thomaz, 2009; Kovalenko et al., 2012; McAbendroth et al., 2005) despite several critiques and warnings (Berntson & Stoll, 1997; Bez & Bertrand, 2011; Halley et al., 2004; Kenkel, 2013). For instance, Thomaz et al. (2008) used D as a proxy for habitat complexity while acknowledging that macrophytes were not true fractals. In the next section, we explain why D is hard to measure and why it should be avoided or used only with great caution.

#### Sources of error in estimating fractal dimension of natural objects and patterns

##### Empirical objects not being true fractals (problems related to quantisation)

**Is the object from which we are trying to estimate** D **close to fractal?** Real‐world objects, as opposed to mathematically idealised objects, cannot be truly fractal. At sufficiently small or large scales, any self‐similar properties of real‐world objects break down. At best, real‐world objects may exhibit close‐to‐fractal behaviour over some range of scales, and it is important to assess if this is true before any attempt at estimating fractal dimension is made.

Even if an object is close to fractal over a range of scales, the possible scales at which we can assess this is limited by data acquisition methods (Kenkel, 2013). Any means of capturing or representing a natural object or surface, whether by pixel‐based digital images, or vector‐based lines and paths, introduces limits on the range of scales in the resulting representation, set by the representation's resolution (the smallest scale captured) and extent (the largest scale).

**FIGURE 3:**
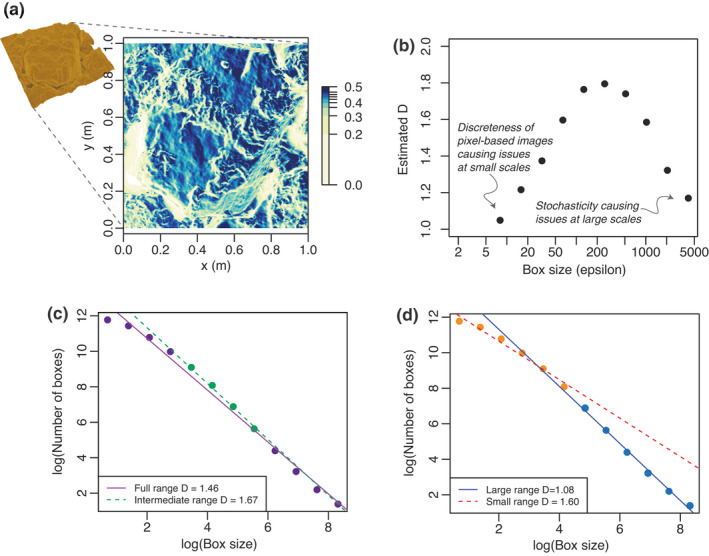
Illustration of the difficulty of measuring fractal dimension (D) from real surfaces. (a) 3D photogrammetry was used to obtain a 1 m × 1 m scan of a rocky intertidal DEM with a resolution of 4096 × 4096 pixels. (b) Box‐counting was applied to estimate the fractal dimension (D) of the DEM (heightmap) across the full range of observed scales (i.e. approximately 3 orders of magnitude). Different D values at different scales were obtained, but because of biases in the methods used to estimate D at small and large scales (see main text), it is not possible to reject the hypothesis that the object is (close to) fractal. (c) Fitting a regression line to all points resulted in an estimated D of 1.46. However, this naive estimation of D across the full range of scales is unreliable (Gonzato et al., 1998, 2000; Kenkel, 2013; see also Box 2). To improve the estimation, we fitted a regression line only across points in the intermediate range of box sizes (green points in part (c)). Estimated D from this portion of the log‐log plot resulted in a D of 1.67. (d) Fitting two separate regressions across the larger and smaller ranges also yields different values of D.

With these technical considerations in mind, we can ask over what range of scales we must measure an object to test whether it is close to fractal (Gneiting et al., 2012; Malcai et al., 1997). Gonzato et al. (1998) suggested that at least 2–3 orders of magnitude are needed (Figure 3), a criterion that most scientific studies of fractality have historically failed to satisfy (Avnir et al., 1998), with some notable exceptions (Bouchaud, 1997; Mandelbrot, 1998). Based on the conservative upper bound of three orders of magnitude, this means that for a 0.001 m (1 mm) resolution digital elevation model (DEM) for example, one would need to capture a minimum quadrat size of 1 m × 1 m at the very least to assess fractality (Figure 2b). Assessment of fractality over such a range of scales is feasible with modern imaging and measurement technology, but most practising ecologists continue to omit this step; in fact, ecologists tend to assume a priori that a natural object is fractal, even though it could also be a multifractal, that is an object requiring different values of D at different scales (Evertsz & Mandelbrot, 1992; Schertzer & Lovejoy, 1989; Seuront et al., 1996), or a complex non‐fractal object. For multifractal objects, alternative metrics including binomial and multinomial measures are available (although these are rarely if ever used in ecology; Evertsz & Mandelbrot, 1992).

##### Measurement error

**Assuming the object is close to fractal, how can we accurately measure** D **?** Before we can estimate D, we need to decide on an appropriate method. Note that testing if an object is close to fractal (see point above) is a separate problem to measuring D itself. Both require a sufficiently large range of scales (Figure 3), but the requirements for measuring D are somewhat less stringent. A variety of methods have been developed to estimate D in different contexts, with some performing better than others, but all are subject to limitations. Examples of methods that have been tested against simulated fractal DEMs and heightmaps (2<D<3) include the triangular prism, isarithm, variogram, box‐counting and variation methods (Klinkenberg & Goodchild, 1992; Lam & De Cola, 2002; Zhou & Lam, 2005).

BOX 2 **Technical issues with using the box‐counting algorithm to estimate fractal dimension and recommendations for mitigating them**

**The box‐counting algorithm**

If the number of boxes of edge length 𝜖 needed to cover a fractal is (𝜖) then mathematically the box‐counting dimension is defined as

(B1.1) $$D=-\lim_{\epsilon \to 0}\frac{\log N\left(\epsilon \right)}{\log\epsilon }$$

Standard box‐counting algorithms estimate D by performing a regression of $\log N\left(\epsilon \right)$ versus ϵ over some practically measurable range of ϵ. We identify four issues with the use of the box‐counting algorithm and make recommendations about how to deal with them.

- **Box‐counting dimension at large scales**

For finite numbers of boxes, where stochasticity due to sampling error can be substantial, the observed value of $N\left(\epsilon \right)$ actually underestimates the true value (Kenkel, 2013). As a result, the box‐counting dimension will tend to be underestimated at large scales, because the gradient of the curve of observed $\log N\left(\epsilon \right)$ versus ϵ will be shallower than the true gradient.

ii **Box‐counting dimension at small scales**

The measured box‐counting dimension will tend to D=1 at very small scales, because any digital representation of a fractal inside a computer will be only a finite‐resolution approximation of a fractal (see Section ‘Sources of error in estimating fractal dimension of natural objects and patterns’). For example, the fractal dimension of the Koch snowflake is $D=\frac{\log4}{\log3}\approx 1.26$, but a typical digital representation of such an object inside a computer is the boundary of a set of pixels, which has dimension D=1, and if we zoom in on it close enough, the box‐counting algorithm will estimate D=1 (the correct value for the digital representation, though not for the underlying idealised fractal object). Similarly, a record of Brownian motion may be mathematically well defined as an object with dimension D=2−H, but a digital representation is a sequence of line segments (Figure S3.1a), which again has dimension D=1.

iii **Box‐counting dimension and regression assumptions**

Even for truly fractal objects, violation of assumptions of the regression methods used to estimate D can lead to biases. A typical method involves first log‐transforming Equation (B1.1) to get

(B1.2) $$\log N\left(\epsilon \right)=c-D\log\epsilon$$

where c is a constant, and then estimating the coefficients c and D via a linear regression of $\log N\left(\epsilon \right)$ on logϵ. The four assumptions of standard linear regression methods are (a) linearity of the true relationship; (b) homoskedasticity of residuals; (c) independence of observations; and (d) normality of residuals. When testing whether an object is truly fractal, all of these assumptions are potentially violated; when measuring D of an object assumed to be fractal, all except (a) are potentially violated (see Supporting Information S3 for details on how to deal them).

iv **Offset and alignment of box grid**

Ideally, the box grid should be aligned to a random origin with the putative fractal being measured. If the box grid is aligned visually to interesting areas of the fractal or if it includes regions outside the fractal, biases in the estimate of D can result (Bouda et al., 2016; Foroutan‐pour et al., 1999; Gonzato et al., 2000; Figure S3.1b). Thus, when using box‐counting the entire box grid should be within the fractal and the offset should be random and not systematically aligned to any particular point (Figure S3.1b). This issue does not apply to 2D heightmaps/DEMs, where the observation window is a subset of a larger putatively fractal region.

**Recommendations**

In view of issue (iv), we recommend that the box‐counting method be applied with random origins and that regions that are not part of the fractal be excluded. In view of issues (i) and (ii), only intermediate box sizes 𝜖 should be used (see Section ‘Sources of error in estimating fractal dimension of natural objects and patterns’). In view of issue (iii), we recommend that the assumptions of regression methods used to estimate D be checked carefully and alternative methods used where appropriate; particular care is needed when using regression to assess whether an object is fractal in the first place.

**FIGURE 4:**
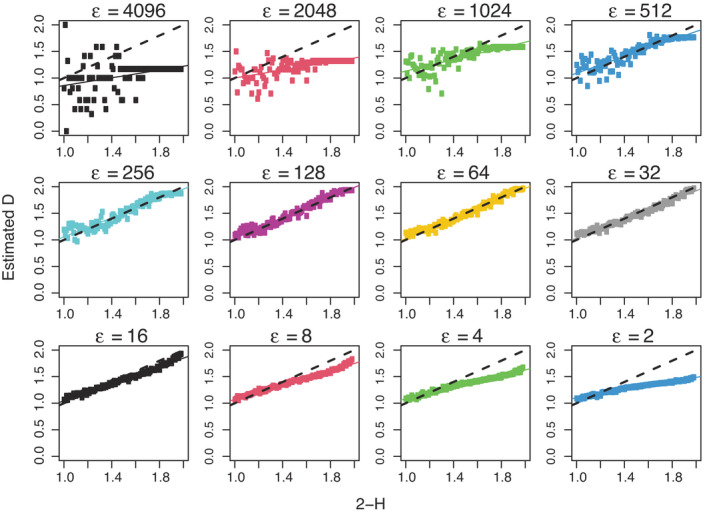
Results of an empirical investigation of the box‐counting algorithm to estimate fractal dimension (D). We generated two‐dimensional binary maps of dimension 4097 × 4097 from a midpoint‐displacement algorithm with values of 𝐻 ranging from 0.01 to 0.99. The horizontal axes show the true fractal dimension D=2−H, where 𝐻 is the Hurst exponent in the midpoint‐displacement algorithm; the vertical axes show the box‐counting estimate of 𝐷 at each box size 𝜖 (panels); the dashed line on each panel is the one‐to‐one line; each point on a given panel is for a single map measured at the corresponding box size ϵ. Our goal was to estimate D assuming that the object was fractal, rather than assess whether the object was actually fractal. Using the box‐counting method with a random origin, the resulting estimates of 𝐷 are low for small 𝜖 (as predicted based on issue (ii); see Box 2) and large 𝜖 (as predicted based on issue (i); see Box 2), but accurate for intermediate ϵ≈32−128 (points are close to 1:1 line). We ran a similar analysis using maps generated from a Gaussian random field algorithm and again found that the results of the box‐counting algorithm were most accurate for intermediate ϵ≈64, although the errors were larger than for the midpoint‐displacement maps (Supporting Information S4).

We will not describe and compare all these methods here, as several previous reviews and simulation studies from other scientific disciplines such as mathematics and statistics have done so (e.g. Barton & La Pointe, 1995; Gallant et al., 1994; Gneiting et al., 2012; Klinkenberg, 1994; Klinkenberg & Goodchild, 1992). Instead, we will cover two methods used broadly in ecology: the box‐counting method (the most widely used method; see Box 2 and Figure 4) and the variation method (an increasingly used method; Dubuc et al., 1987; Parker, 1997; see also Supporting Information S2).

The box‐counting method is affected by four main technical issues: (i) errors at large scales due to stochasticity associated with small sample sizes, which induce a negative bias in D; (ii) errors at small scales due to finite resolution, which also induce a negative bias in D; (iii) errors due to violations of regression assumptions; and (iv) errors due to the inclusion of non‐fractal regions and to the offset of the box grid not being random but deliberately aligned with visually prominent regions of the fractal. These issues are explained in more detail in Box 2 and Figure 4.

The ideal procedure for testing any fractal dimension estimation method is to apply it to simulated objects of known D. Some studies have instead compared methods using empirical 1D profiles (e.g. Breslin & Belward, 1999) and 2D surfaces (e.g. Klinkenberg & Goodchild, 1992), but it is not possible to assess accuracy in these cases because the true D values are unknown. In Supporting Information S2, for instance, we critically evaluate a study (Torres‐Pulliza et al., 2020) whose claims to have developed a unified geometric basis for surface habitat complexity and biodiversity evaporate when their framework is tested against simulated data with known D. To compare five different methods of estimating D, Zhou and Lam (2005) tested them against simulated fractal DEM surfaces (i.e. 2<D<3). Some estimators performed better than others, but none produced reliable estimates of D. However, their approach was based on a naïve box‐counting method that uses the full range of spatial scales, ignoring issues (i) and (ii) above, that is discreteness of the pixel image at very small scales and stochasticity at very large scales (see also Box 2). Limiting the range of scales addresses these issues and leads to better estimates of D (Dubuc et al., 1989; Gneiting et al., 2012; Kenkel, 2013; Liebovitch & Toth, 1989; Pruess, 1995; Figure 3; Box 2). However, discarding information at small and large scales means lower statistical power, and to mitigate this an even broader range of scales of measurement is needed.

**FIGURE 5:**
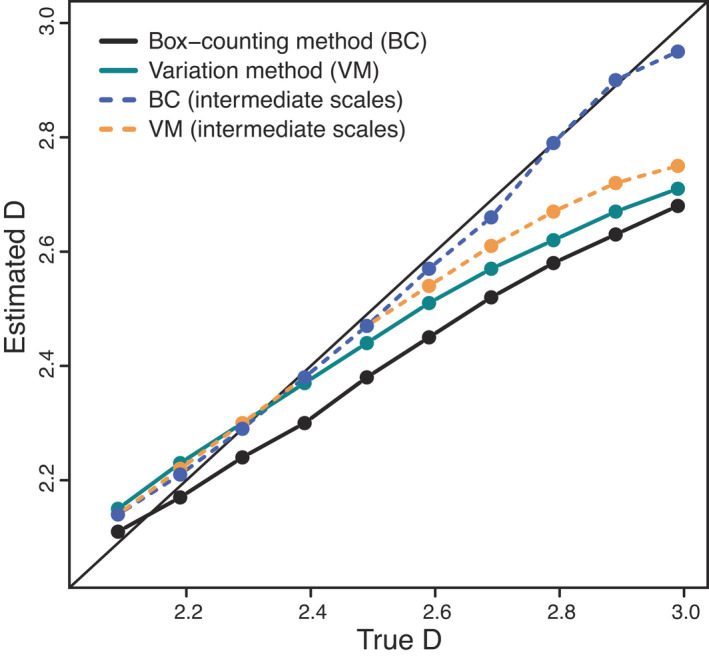
Estimates of fractal dimension D for two‐dimensional binary fractal (fBm) maps of dimensions 4097 × 4097 generated by the midpoint displacement algorithm (e.g. Figure 2a). The Hurst exponent H was varied systematically across simulated maps. We applied the box‐counting and variation methods to estimate D (averaged from 100 maps for each 𝐻 value). The horizontal axis corresponds to the true fractal dimension of the simulated maps (D=2−H); the vertical axis corresponds to the estimated 𝐷 using each method. The solid line is the 1:1 line. Box‐counting at intermediate scales produced estimates closest to the 1:1 line.

We conducted a test of four different methods of estimating D to test how each performed in recovering the true D of simulated fractal maps (Figure 5). The four methods were: (1) box‐counting, using naïve estimation over the full range of available scales; (2) the variation method, also over the full range of available scales (Dubuc et al., 1987; also the approach used by Torres‐Pulliza et al. (2020); refer to Supporting Information S2 for more details); (3) box‐counting over intermediate scales; and (4) the variation method over intermediate scales.

Consistent with previous studies (e.g. Panigrahy et al., 2019; Zhou & Lam, 2005), our results show that low values of D were overestimated and high values of D were underestimated by both the naïve box‐counting and variation estimators (Figure 5). The box‐counting method restricted to intermediate scales resulted in better estimates of D, though there was still some bias (Figure 5). The variation method at intermediate scales performed only slightly better than the naïve variation method. It is important to emphasise that these difficulties arise even under our idealised conditions where the measured object is generated from a perfectly fractal process and there are no systematic sources of error, other than the inevitable digitisation errors.

#### Limitations of fractal dimension

Assuming an object is close to a true fractal and that D can be measured accurately (itself a dubious prospect—see previous subsection), the question becomes whether D is correlated with biological variables of interest. For species richness, the results are equivocal. Although some studies have found positive associations between D and richness, the variance explained is usually low (Table S1). For example, in one study of coral reefs, D only explained 4.6% of the variation in coral species richness (Torres‐Pulliza et al., 2020) and in another study of seabirds it explained 1–6% of the variation in species richness (Hashmi & Causey, 2008). Less frequently, there have been reports of high correlations between D and richness (Table S1); for instance, Dijkstra et al. (2017) showed that D explained 71% of the variation in meso‐invertebrate richness on seaweeds.

Besides species richness, a number of other biological variables of interest, such as body size and density of individual organisms, exhibit correlations with D (i.e. Beck, 1998; Gunnarsson, 1992; Hills et al., 1999; Jeffries, 1993; McAbendroth et al., 2005; Morse et al., 1985; Schmid et al., 2002; Shorrocks et al., 1991; Taniguchi & Tokeshi, 2004). For example, while McAbendroth et al. (2005) did not find any relationship between D and richness of small invertebrates in complex macrophytes, they found a positive relationship between D and invertebrate density. Similarly, Dibble and Thomaz (2009) measured D from images of 11 species of tropical and temperate macrophytes and found weak correlations with measured densities of odonates and annelids, although they did not measure species richness. As a side note, D is sometimes incorporated into other compound indices used as proxies for habitat complexity (e.g. ‘stand structural complexity’ or SSCI; Ehbrecht et al., 2021). Such compound indices, however, by nature obscure rather than illuminate the effect of D on diversity and other biological variables. High correlations could be due to variables other than D included in an index.

In possible defence of D, it could be argued that the observed correlations with diversity are mostly low because of errors in estimated D due to the aforementioned measurement problems (Section ‘Sources of error in estimating fractal dimension of natural objects and patterns’). However, measurement errors cut both ways: it is also possible that even the occasional high correlations with diversity currently observed are illusory, due to something other than D being inadvertently measured. It could also be argued that if measured D correlates with diversity, it could be a useful metric regardless of whether the object is truly fractal. The retort here is that such a metric would be useful only if we also specify the scale at which D must be measured—in which case the use of the term ‘fractal dimension’ is misleading because there is no requirement for any degree of self‐similarity. For example, Beck (1998) found weak positive correlations between D (estimated from ten 300 mm transects within 0.25 m^2^ quadrats with a resolution of 5 mm) and gastropod species richness in rocky intertidal and mangrove habitats, but the photogrammetric method used captured less than two orders of magnitude of scale, and it is likely that different values of D and thus different correlations with diversity would be obtained at other scales. We illustrate this in Supporting Information S5 using our own data from an intertidal community.

Returning to our main question about how to measure complexity, a technical problem with fractal dimension is its non‐monotonic relationship to complexity. Objects with integer fractal dimension, such as a plane (D=2) and a cube (D=3), are clearly not complex. In between these integer values, complexity likely obeys a hump‐shaped (unimodal) relationship to D, increasing at first and then decreasing. But there is no consensus on the precise value of D at which complexity peaks (Flores, 2022; Gao et al., 2007; Kak, 2021; Palmer, 1992). Nevertheless, in many situations in ecology, values of D have a small decimal component (e.g. D values around 2.1 or 2.2 are common), and the practical implications of this technical issue may be minimal: assuming (generously) that all the other issues with D can be resolved, ecologists can safely assume that increasing D implies increasing complexity.

#### Verdict on fractal dimension

Although fractal dimension D is mathematically well defined, in principle broadly applicable and independent of area, it is difficult to measure in practice (i.e. it satisfies our criteria (1), (2) and (4) in Section ‘Ideal qualities of a metric of complexity’ but fails criterion (3)). Real‐world objects cannot be truly fractal, and even for objects that are close to fractal over a range of scales, it is very difficult to ascertain this in practice (Figure 3) and to estimate D accurately (Figure 5). Measurement issues can be mitigated to some degree by using higher resolution imagery with large spatial extent, and by estimating D only at the intermediate scales captured (Box 2, Figure 4). In the light of these issues, we do not strongly recommend D as a metric of surface or habitat complexity in ecology. Even if the technical issues were resolved, the evidence to date suggests that D is not highly correlated with diversity. Although this may simply indicate that complexity has little effect on diversity, another possibility is that D does not actually measure the aspects of complexity that are relevant to organisms. This latter possibility motivates the search for other complexity metrics.

### Rugosity

Rugosity (R) is another common measure of structural complexity in ecology. When applied to 2D surface habitats, it is a relative measure of the amount of surface area within a given planar area (i.e. a parallel projection of a surface onto a plane). The formula for true rugosity ($R^{*}$) of a surface with square projection is

(2) $$R^{*}=\frac{A}{L^{2}},$$

where A is surface area, and L is the edge length of the projected square. When applied to 1D profiles, it measures the total length of the profile's contour ($L_{m}$) over a fixed distance or linear extent (L):

(3) $$R^{*}=\frac{L_{m}}{L}.$$

Accuracy in the estimation of $R^{*}$ depends on the approaches used to estimate A (Equation 2) or $L_{m}\;$(Equation 3), which can be sensitive to the resolution of the measured object, particularly if the object has fractal qualities. When applied to transects of a 2D surface, the estimate from Equation (3) gives results consistent with Equation (2) provided that the surface topography is isotropic. Rugosity is sometimes called topographic or surface roughness in the ecological literature (e.g. Figueira et al., 2015; Frost et al., 2005).

#### Pitfalls of using rugosity as a metric of complexity

One standard way of estimating R is to measure the total length of a 1D profile (e.g. using a profile gauge or a chain with links draped over the physical surface of a habitat) and divide that by the linear extent as in Equation (3) (Friedman et al., 2012; Luckhurst & Luckhurst, 1978; McCormick, 1994; Risk, 1972); the resolution of this approach is the width of each chain‐link or the distance between the pins of a profile gauge.

Another way of estimating R is to measure 2D surface area directly from a digital model and then to divide this by planar area (Equation 2). This approach should yield more precise estimates of R than averaging across multiple 1D profiles (e.g. Bayley et al., 2019; Young et al., 2017), because the entirety of the surface is being used. In other words, if the 2D approach is feasible, which it now almost always is with digital technology, the 1D method is redundant. Care should also be taken to minimise potential sources of error (see next subsection).

#### Sources of error in estimating rugosity of natural objects and patterns

##### Measurement error (choice of approximation method)

As mentioned in Section ‘Pitfalls of using rugosity as a metric of complexity’, the choice of method used to approximate total surface area (A) or profile length $\left(L_{m}\right)$ (Equations 2 and 3) can influence the accuracy of R estimates across a range of scales. Given the simplicity, the cost and time effectiveness, and the accuracy of modern computer‐based methods (Du Preez, 2015; Young et al., 2017), it is likely that traditional field‐based approaches will gradually be phased out. While R estimates from 3D models may still be prone to some error resulting from the influence of slope and aspect angle (Figueira et al., 2015; Porter, 2019), Friedman et al. (2012) and Du Preez (2015) demonstrated how this methodological issue can be corrected for using a plane of best fit.

##### Choice of resolution

**FIGURE 6:**
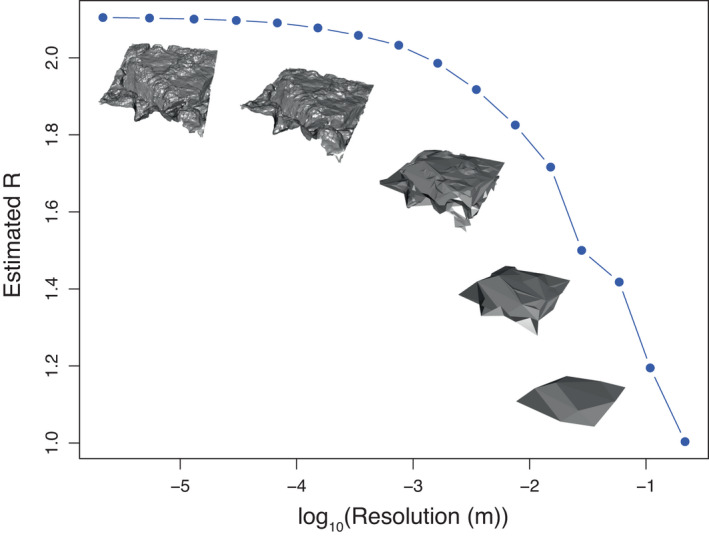
Illustration of how estimated rugosity R of a 1 m × 1 m rocky shore DEM changes with decreasing resolution (or increasing granularity). Since the planar area was 1 m^2^, R effectively represents total available surface area at a given resolution in square metres.

Regardless of the estimation approach, the value of R fundamentally depends on the resolution at which the observation was made (i.e. complexity varies with the scale at which it is measured; Porter, 2019; Yanovski et al., 2017), with R increasing with increasing resolution (Figure 6). A given species may respond to R at some scales but not others. For instance, gastropods on rocky intertidal substrates may only perceive microhabitats and respond to R measured at scales closer to their body sizes (millimetres to centimetres) rather than R measured at scales of metres. Such observations have been made in the field: for example, both fish on coral reefs and amphipods in benthic algae have been found to associate with greater rugosity at scales comparable to their body sizes (Hacker & Steneck, 1990; Wilson et al., 2007). Thus, to compare R of different habitats and across ecosystems with different suites of species, we need to measure R across a wide range of resolutions. Doing so is feasible with computer‐based approaches, though not with field‐based approaches. This may explain why traditionally few studies in ecology have actually measured R across a wide range of resolutions (e.g. Porter, 2019; Yanovski et al., 2017) and even fewer have related their R estimates back to measured ecological responses (but see Porter, 2019).

#### Limitations of rugosity

##### Confounding effects of area

Although existing evidence suggests that rugosity R is often strongly positively correlated with species diversity (e.g. McCoy & Bell, 1991; Risk, 1972; Torres‐Pulliza et al., 2020), it is possible that this simply reflects the greater surface area available at higher R (the fundamental species–area principle; Lawton, 1999), rather than other aspects of habitat structure that we might typically associate with complexity (see next subsection). Rugosity fails our criterion (4) that a good metric of complexity should be decoupled from the effects of area (see Section ‘Ideal qualities of a metric of complexity’). If a landscape with projected area $L^{2}$ has rugosity $R^{*}$ at a given resolution, then the surface area available to organisms at this resolution is $A=L^{2}R^{*}$ (from Equation 2). The power‐law species–area relationship then predicts that $\log S=c+z\log A=c+z\log L^{2}+z\log R^{*}$, whence it is clear that regressing S on rugosity is likely to yield positive correlations due to area effects alone. While this does not negate the role of R as a useful metric or predictor of diversity, it fails to address the specific goal of establishing a metric of complexity decoupled from area.

##### Failure to account for relevant habitat structure

**FIGURE 7:**
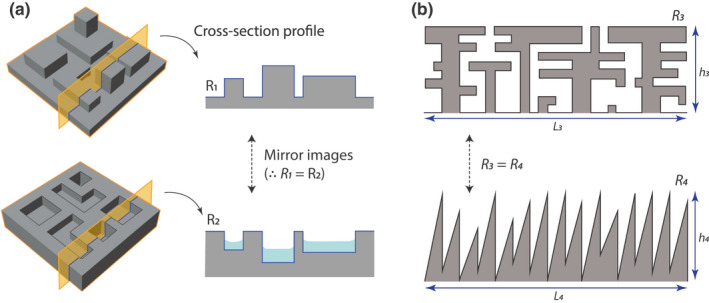
Illustrations of the limitations of rugosity as a complexity metric in ecology. (a) Two hypothetical surfaces with identical surface area and R values but different geometrical features (i.e. ‘component types’), with different consequences for biodiversity. In an intertidal context, for example, the bottom surface will retain more water during low tide and provide refugia for a variety of benthic species. Analogous examples could be constructed for other systems, for example, macroscopic terrestrial landscapes where peaks and valleys of different altitudes provide habitats for different species. (b) Two hypothetical 1D profiles with identical R values, linear extents (L) and heights (h) but different geometrical features: the top profile has overhangs and interstitial spaces and is arguably more complex, and may harbour a greater diversity of species.

The use of R as a metric of complexity overlooks important aspects of habitat structure such as the shape and configuration of habitat elements (Beck, 2000; Figure 7). Two habitats can have very different geometric configurations, with probable different consequences for biodiversity, but have the same R value (Figure 7a; see also Loke & Todd, 2016). In addition, the measurement of R in practice often ignores habitat components associated with complexity, such as overhangs and interstitial spaces (e.g. Figure 7b), as most remote sensing methods are not able to capture what is within or under objects.

#### Verdict on rugosity

Rugosity R is always well defined (for a given resolution) and much easier to measure than fractal dimension. It also appears to be more highly correlated with diversity. However, being strongly confounded with surface area, it may be a poor metric of complexity *per se* and, in addition, it is relevant only to certain types of objects (in terms of our criteria in Section ‘Ideal qualities of a metric of complexity’, it satisfies (2) and (3) but fails (1) and (4)). Rugosity fails to capture aspects of habitat structure that would typically be associated with complexity (e.g. Figure 7b). While we recommend the use of R (or area itself) to characterise surface habitats, we warn that it is at best an incomplete descriptor of complexity, which may nevertheless be correlated with diversity across a range of scales and systems. We urge future studies using R to acknowledge the effects of area on diversity, and should area itself be used as a metric that it be explicitly stated as such and not labelled as ‘complexity’. A sensible approach would be to include surface area as a covariate in any regression of species richness S on rugosity (with all variables logarithmically transformed: logS=alogA+blogR+c), to test the effects of rugosity independent of those of area.

### Other geometric metrics of complexity

In this section, we give an overview of other geometric complexity metrics used in ecological applications (Figure 1b).

Height variation is occasionally used as a metric of habitat complexity (Figure 1b). Height variation can be measured simply as the range of surface heights. At large scales, some measure of elevation (i.e. height of the Earth's surface above a particular geographic reference point) or height variation is often used to describe the ‘topographic complexity’ of terrain—perhaps due to the availability and ease of extracting the necessary information directly from georeferenced maps—and topographically complex regions (e.g. regions with high relief created by tectonic uplift and geological erosion) have been found to feature disproportionately greater ecological, taxonomic and species diversity (Badgley, 2010; Badgley et al., 2017). Another index, ‘terrain/topographic ruggedness’ is computed from a heightmap as the root‐mean‐square of differences in height values between a focal cell and its eight surrounding cells, averaged across all focal cells (Riley et al., 1999).

Less commonly, complexity may also be quantified in terms of surface angle variation. Surface angle or slope refers to the change in vertical distance over the change in horizontal distance and may be expressed in terms of vectors and angles. ‘Vector dispersion’, which can be extracted from 3D models and digital elevation models, is sometimes used as a metric of habitat complexity as it expresses the uniformity of surface angles at a given resolution (see Grohmann et al., 2010; Young et al., 2017 for more details). Low vector dispersion indicates smooth surfaces, whereas high vector dispersion indicates rough surfaces. Previously discussed sources of error in estimating R apply to measures of both height variation and vector dispersion (see Section ‘Sources of error in estimating rugosity of natural objects and patterns’).

Relative to the popular measures D and R, fewer studies use variations in height, slope and angles as metrics of geometric complexity (Figure 1b), and the evidence linking them to diversity at local scales remains equivocal and difficult to generalise (e.g. contrasting environmental gradients between marine vs. terrestrial landscapes). Carleton and Sammarco (1987) found that vector dispersion and slope explained 36% and 43% of variation in coral genus richness at fine (millimetre to centimetre) scales. But McCormick (1994) found that vector dispersion, slope and surface height explained only 19%, 8% and 2%, respectively, of the variation in fish species richness on coral reefs at coarser (metre) scales, and in a recent study conducted at similar scales at the same location, Torres‐Pulliza et al. (2020) found that surface height range explained just 4% of the variation in coral species richness.

These other metrics are also likely confounded with surface area to some degree (e.g. a landscape with higher vector dispersion will tend to have higher surface area), and so to test the effects of complexity independent of area, a multiple regression approach as outlined at the end of Section ‘Rugosity’ may be appropriate.

### Information‐based metrics of complexity

In the previous sections, we have covered metrics of geometric complexity and their limitations. In some cases, surmounting these limitations may involve going beyond purely geometric notions of complexity (e.g. Figure 7a). Informational complexity metrics are a promising alternative (Figure 1b). Informational metrics of complexity are in many cases correlated with geometric metrics of complexity, but can describe a broader variety of objects and may provide a more unified perspective.

Informational complexity is the amount of information needed to encode and describe an object or system. A common measure of informational complexity is entropy (Rissanen, 2007) or, more specifically, Shannon entropy (Shannon, 1948). Take a set of N observations on a random variable X that has k possible outcomes, and where the probability to observe $X=x_{i}$ is $p_{i}$ for i=1,…,k. The Shannon entropy of a single observation on X is

(4) $$H=-\sum_{i=1}^{k}p_{i}\log_{2}p_{i}$$

with the summand taken to be zero when $p_{i}=0$. The entropy of the entire set of N observations is NH, and represents the number of bits of information required to describe the observations. The maximum value of entropy occurs when the outcomes are equally likely, that is $p_{i}=1/k$ for all i, in which case from Equation (4) we have $H=\log_{2}k.$ The minimum value of entropy, H=0, occurs when $p_{j}=1$ for some j (and thus $p_{i}=0$ for all i≠j) (Witten, 2020). In general, objects with higher degrees of randomness have greater entropy than those with repeating elements and other regularities.

Informational complexity can be measured in other ways, such as algorithmic information content (Kolmogorov, 1983), statistical complexity (Crutchfield & Young, 1989), among many others (see also Badii & Politi, 1997; Crutchfield, 1994; Gell‐Mann & Lloyd, 1996, 2004; Lloyd & Pagels, 1988; Shalizi & Crutchfield, 2001). Different metrics capture different features of complex systems; statistical complexity, for instance, captures aspects of complexity that are distinct from pure randomness (Ladyman & Wiesner, 2020), but as these other metrics were developed in other scientific fields, they have rarely been applied in ecology (see Section ‘Future research’).

To measure the informational complexity in an ecological application, one first must define what constitutes a relevant element in the system, a potentially non‐trivial exercise involving some degree of subjectivity. Examples of relevant elements could be features on a landscape map (e.g. habitat patches, land cover classes, spatial points, lines, networks) or features within habitats (e.g. different kinds of microhabitats). Entropy and other metrics of informational complexity can then describe the variability and arrangement of these elements.

**FIGURE 8:**
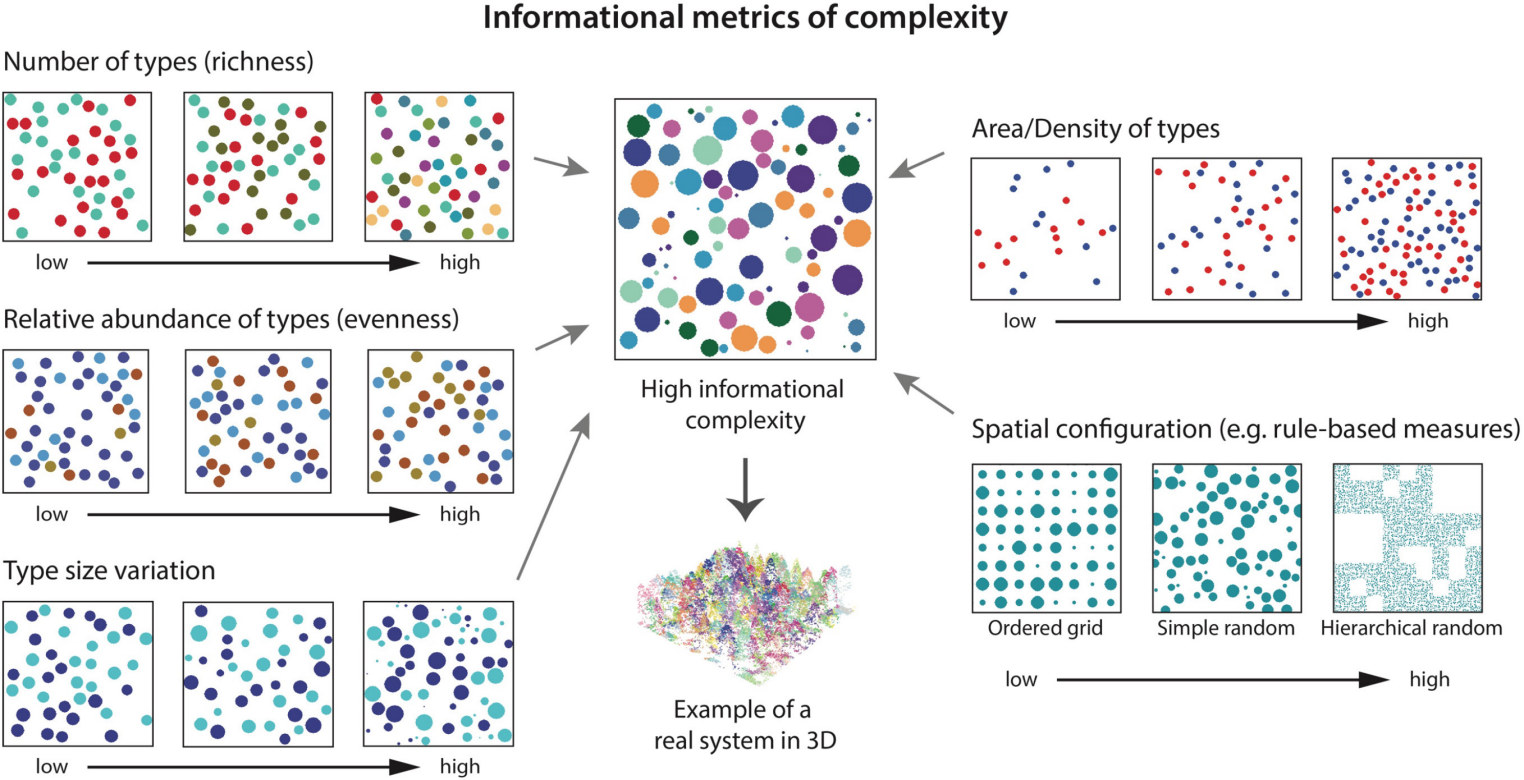
Examples of how discrete elements in a system may be altered to manipulate its informational complexity. Different types of elements are represented by circles of different colours. For instance, the spatial configuration of elements on a 2D map may be defined by some rule (e.g. ‘ordered grid’, ‘simple random’ or ‘hierarchical random’); the more ordered a system is, the lower its informational complexity as less information is required to describe it. These rules fit with intuitive notions of complexity; for example, an oil palm plantation (ordered; low density of types, number of types, and size variation) would be considered less complex than a tropical forest (random; high density of types, number of types, and size variation as depicted in the 3D example at centre‐bottom).

Ecologists' intuitions about complexity are broadly congruent with informational complexity: disorder and randomness lead to habitats with more niches and thus more species. This is reflected in the way many ecologists have tried to quantify or recreate habitat complexity and spatial heterogeneity using metrics such as the density, number of different component types and their variability (Figure 1b), which are essentially different ways in which the informational content of an object or system can be altered (Figure 8; e.g. Beck, 2000; Eriksson et al., 2006; McCoy & Bell, 1991; see Loke et al., 2014, 2015 for more details on how these variables alter the informational complexity of a system).

Many local‐scale ecological experiments manipulate informational complexity (Figure 1b), by controlling the amount, variability or spatial positions of select elements in a system (Figure 8). Informational complexity largely overlaps with the concept of ‘heterogeneity’ in ecology. For example, Loke et al. (2017) manipulated complexity by varying the size range (widths and depths) of different microhabitat elements (pits and grooves) within a given planar surface while standardising for area, and Cardinale et al. (2002) manipulated benthic stream substrate heterogeneity by altering the range of particle sizes while holding the median size constant.

As an operational framework, information‐based complexity has several advantages: it can be applied to multiple classes of objects and systems across scales (our criterion (1) in Section ‘Ideal qualities of a metric of complexity’), it can be easily measured (criterion (3)), and it allows the effects of area and density to be disentangled from other aspects of complexity (criterion (4)) (see also experiments by Ben‐Hur & Kadmon, 2020b; Loke & Todd, 2016; Loke et al., 2019). With complexity being the broader concept that subsumes heterogeneity, information‐based complexity accounts for heterogeneity and other types of complexity. However, one limitation of informational complexity is that it relies on somewhat subjective criteria for selecting what constitutes an element in the system (i.e. criterion (2) in Section ‘Ideal qualities of a metric of complexity’).

## FUTURE RESEARCH

Ultimately, the goal of having a metric of habitat complexity and spatial heterogeneity in ecology is to understand the forces that structure biodiversity and other community properties. A metric may be mathematically well defined (like D), but if it is hard to measure or does not capture aspects of complexity relevant to organisms, it has little relevance. We may, through other disciplines such computer science and physics, discover and develop different candidate metrics, but only empirical work can reveal the relevance and importance of any metric in ecology.

A word of warning is that in any given system the forces structuring diversity may actually be simple, being driven, for example, by available surface area, or being associated with just one idiosyncratic aspect of complexity, such as pits or overhangs in intertidal communities (Figure 7). Furthermore, complexity is not a fixed property of the environment because organisms themselves can create complexity by forming habitats. Ecological systems are complex systems that are dynamic and so quantifying habitat complexity at specific points in time (compressing the dimension of time) may also leave out important temporal aspects of complexity. In view of all this, efforts to come up with a universally applicable metric of complexity may be futile.

Nevertheless, at least for specific systems, the measurement of complexity continues to be an important problem in ecology for both basic and applied reasons. Given that we still do not have metrics that can consistently explain a large fraction of the variation in species diversity, there is cause to keep looking, even if such an endeavour yields no metric better than R (Section ‘Limitations of rugosity’). For example, measures of informational complexity that incorporate correlations between elements may better characterise the heterogeneity of ecological systems than do simple entropy measures (Xiong et al., 2017). Explorations into information‐based measures of complexity may also potentially offer novel ways to characterise niche space, which may ultimately yield insights into the basis of community assembly and species coexistence. We encourage closer collaborations between field ecologists, experimentalists and theoretical ecologists to see what structural metrics of complexity are predicted to be important for explaining biodiversity and consequently ecosystem function in systems of interest. Our lists of essential and desirable qualities that a good metric should possess in Section ‘Ideal qualities of a metric of complexity’ serve as guidelines for future work.

### On the rise of remote sensing technologies in ecology

Remote sensing technologies are increasingly accessible and widely applied in ecology (D'Urban Jackson et al., 2020; Kerr & Ostrovsky, 2003). Tools for 3D mapping such as lidar, radar, sonar and techniques for processing remotely sensed data such as structure‐from‐motion photogrammetry have now facilitated, with relative ease, the reconstruction of high‐resolution maps of vast areas of different ecosystems from which all kinds of habitat–structure variables can be extracted (Pettorelli et al., 2014; Turner et al., 2003). For instance, in forests a large suite of putative complexity variables, such as canopy density, heights, composition, or below‐canopy tree and shrub layers can be extracted from lidar data (Müller & Brandl, 2009), and these have been used in species distribution modelling to predict patterns of species diversity for conservation planning and prioritisation (Zellweger et al., 2013). Supplemented with calibration data painstakingly collected by field ecologists through ground surveys, they can be used, for example, to identify hotspots of biodiversity in remote and hard‐to‐access areas in both terrestrial (e.g. Schut et al., 2014; Turner et al., 2003) and marine environments (e.g. Saunders et al., 2020).

Given the rapid advancement of these technologies and adoption by ecologists, it is important to appreciate the interpretive and practical issues with common metrics of complexity such as D and R (and their variations). For instance, while it may be easy to estimate D via box‐counting from 3D models generated from remotely sensed data, or to assume that it is reflecting our intuitive notions of ‘habitat complexity’ in a scale‐free manner, these assumptions may quickly legitimise arbitrary interpretations of the effects of ‘complexity’ on diversity and impede our understanding of the interaction between complexity and diversity. Thus, while we acknowledge all the benefits that remote sensing technologies will bring to the study of habitat complexity in ecology, we urge caution when it comes to interpreting complexity measures, in particular D.

## APPLIED SIGNIFICANCE

Strategies in applied ecological fields such as conservation biology, restoration ecology and ecological engineering often involve creating or restoring habitat complexity and spatial heterogeneity in fragmented, degraded or simplified environments (Falk et al., 2006; Firth et al., 2016; Gardner et al., 2007; Lindenmayer & Fischer, 2007). Such implementations fall within the International Union for Conservation of Nature (IUCN) framework for nature‐based solutions to societal challenges, specifically under the ecosystem restoration and infrastructure‐related approaches (Cohen‐Shacham et al., 2016). With the rise in remote sensing technologies (Section ‘On the rise of remote‐sensing technologies in ecology’), the idea of translating some metric of habitat complexity (e.g. obtained from scanning diverse or pristine natural environments) into real‐world solutions is tantalising (Calders et al., 2020; Ferrari et al., 2021). These solutions range from replanting or introducing trees and habitat‐forming species to adding physical elements to manipulate habitat structure in both natural and urban environments (Morris et al., 2019; Palmer et al., 2010). Whatever the means, the idea of recreating some level of complexity in a degraded system is largely based on the assumption that this will mimic and recreate niches for organisms, leading to increased levels of biodiversity and ecosystem services.

Complexity metrics are also being used as success indicators for restoration programs. It has been suggested that techniques such as photogrammetry will improve restoration success by enabling the measurement of complexity with ‘unprecedented accuracy’ (Ferrari et al., 2021). Again, this premise is built on the assumption that we already have reliable, accurate metrics of complexity; but, unfortunately, we do not. Simply knowing the required rugosity (R) or fractal dimension (D) of an artificial habitat, for instance, leaves a lot of room for uncertainty regarding the key aspects of design that may be highly relevant to biodiversity (Figure 7). In landscape ecology, it has been suggested metrics such as D may be frequently adopted simply because they are easily generated through landscape analysis software (With, 2019).

Given global rates of environmental change, including habitat loss and climate change, more needs to be done to understand the link between complexity and diversity. Ultimately, success in rebuilding or restoring ecological complexity will require metrics that are straightforward to measure and are correlated with ecological variables of interest, which in turn will depend on a deeper understanding of how habitat complexity begets diversity. The best metrics may be system‐specific rather than universal.

## CONCLUSIONS

There is no consensus in ecology over the definition of complexity or how to measure it. While some metrics of complexity are used more frequently used than others, none is without limitations (Figure 1b; see Section ‘Metrics of complexity’). The terms ‘complexity’ and ‘heterogeneity’ are often used indiscriminately or as a substitute for some aspect of species’ niche requirements (Figure 1a), perpetuating confusion over which aspects of complexity are actually influencing diversity. Addressing the measurement of complexity will allow ecologists to leverage the remote sensing revolution and study complexity–diversity relationships with more precision (see Section ‘Future research’). Targeted system‐specific efforts may initially be most fruitful, and may eventually reveal the bigger picture of how complexity affects diversity in different systems and at different scales, bringing broad benefits for basic and applied ecology.

## AUTHORSHIP

LL contributed ideas, provided data, performed analyses, wrote the first draft of the manuscript and led study. RC contributed ideas, performed analyses, edited the manuscript. All authors contributed substantially to revisions.

### PEER REVIEW

The peer review history for this article is available at https://publons.com/publon/10.1111/ele.14084.

## Supporting information


Appendix S1
Appendix S2
Appendix S3
Appendix S4
Appendix S5

## Data Availability

The data and code supporting these results are available in the Zenodo repository: https://doi.org/10.5281/zenodo.6640505.
