# Supplementary material for: Measuring habitat complexity and spatial heterogeneity in ecology
Source: Ecol Lett. 2022 Aug 17;25(10):2269–88. doi: 10.1111/ele.14084 (PMC9804605; doi:10.1111/ele.14084)
Supplement: Supplementary file 1 — Appendix S1 Appendix S2 Appendix S3 Appendix S4 Appendix S5 [file ELE-25-2269-s001.pdf]

Supporting Information for:

Measuring habitat complexity and spatial heterogeneity in ecology

Lynette H. L. Loke[1,*], Ryan A. Chisholm[2]

[1]School of Natural Sciences, Faculty of Science and Engineering, Macquarie University, North Ryde, NSW, 2109, Australia.

[2]Department of Biological Sciences, National University of Singapore, Singapore 177558.

*Corresponding author: lynetteloke@gmail.com

Appendix S1: Details of bibliographic analysis ———————————— 2

Appendix S2: Case study of Torres-Pulliza et al. (2020) ———————————— 5

Appendix S3: Supplementary information for Box 2 ———————————— 10

Appendix S4: Supplementary results of empirical investigation in Figure 4 ———— 12

Appendix S5: Empirical demonstration of the limitations of D ———————————— 13

Appendix S1: Details of bibliographic analysis

Our bibliography was first generated in Clarivate Analytics Web of Science (WoS) using the following search terms (ecolog* AND "habitat complexity" OR "spatial heterogeneity" OR "habitat structure" OR "habitat heterogeneity" OR "structural complexity"). We selected papers over the last 60 years from 1961 to 2021. This generated a total of 27,864 articles. Given the considerable number of articles returned, we then we performed a bibliometric analysis with the Bibliometrix R package (Aria & Cuccurullo, 2017) to identify any potential conceptual structures that may exist. To do this, the scope of our bibliography was reduced by selecting the following ecological journals: Ecology, Ecology Letters, Landscape ecology, Ecological Monographs, Oikos, Journal of Applied Ecology, Journal of Ecology, American Naturalist, Methods in Ecology and Evolution, Nature Ecology and Evolution; we also complemented the set with articles from the following multidisciplinary journals: Proceedings of the National Academy of Sciences of the USA (PNAS), Proceedings of the Royal Society B Biological Sciences, Nature Communications, Science Advances, Nature, and Science. Through this process, a relevant set of 1374 articles were generated and their metadata (title, author list, journal, keywords, year of publication, abstract, text and reference list) were exported. The conceptual structure of our bibliographic collection was analyzed using a co-occurrence network (which reveals links between keywords based on co-occurrence in the articles) and a multiple correspondence analysis (which reveals clusters based on the keywords metadata). Following the results of our bibliographic analysis, we further reduced our scope by selecting only recent articles (over the last two decades from 2000 to 2021) using the search terms (ecolog* AND "habitat complexity" OR "structural complexity" OR "spatial heterogeneity"). This returned 1002 articles from which we selected the top 150 articles from the following ecology journals: Ecology, Ecology Letters,

Ecological Applications, Journal of Ecology, Journal of Applied Ecology, Oikos, Nature, Science, American Naturalist, Ecological Monographs, Journal of Biogeography, Ecography and Scientific Reports, and complemented this selection with the top 50 relevant results generated by Google Scholar. This resulted in a manageable set of 136 relevant articles. Papers were downloaded and read individually to generate counts for each metric (i.e. Figure 1b). Of all the articles that used fractal dimension (D) as a metric of complexity, we checked whether D was correlated with species richness (S) and abundance (N) (Table S1.1).

Table S1.1 Recent ecological papers (published after 2000; n = 39) that measured fractal dimensions (D). Only 14 of the 39 papers correlated D with species richness (S), and 15 with individual abundance (N). Out of the 14 papers that correlated D with S, the majority found weak relationships.

Reference	*S*	*N*	R^2 (with S)	Strength	Direction
Attrill et al., 2000	✓	✓	Not provided	Weak	Negative
Bailey et al., 2004	-	-	NA	NA	NA
Beck, 2000	✓	✓	NA	NA	Positive
Bodmer et al., 2021	-	✓	NA	NA	NA
Bouda et al., 2016	-	-	NA	NA	NA
Bué et al., 2019	✓	✓	NA	NA	Positive
Burrows et al., 2009	-	-	NA	NA	NA
Carsartelli & Ferragut, 2018	✓	✓	NA	NA	Positive
Commito & Rusignuolo, 2000	-	-	NA	NA	NA
Dibble & Thomaz, 2009	-	✓	NA	NA	NA
Dijkstra et al., 2017	✓	✓	0.71	Strong	Positive
Duarte et al., 2020	✓	-	0.001/0.004	Weak	Positive
Frost et al., 2005	-	-	NA	NA	NA
Fukunaga et al., 2020	-	-	NA	NA	NA
Kalacska et al., 2018	-	-	NA	NA	NA
Hashimi & Causey, 2008	✓	-	0.01/0.06	Weak	Positive
Johnson et al., 2003	✓	-	0.42/0.16	Both	Positive
Kamal et al., 2014	-	-	NA	NA	NA
Kostylev et al., 2005	✓	✓	0.53/0.32/0.46	Strong	Positive
Kostylev & Erlandsson, 2001	-	✓	NA	NA	NA
Kovalenko et al., 2010	-	-	NA	NA	NA
Mancinelli et al., 2007	-	✓	NA	NA	NA
Marsden et al., 2002	-	-	NA	NA	NA
McAbendroth et al., 2005	✓	✓	0.04	Weak	Positive
Pascoe et al., 2021	-	-	NA	NA	NA
Reichert et al., 2017	-	-	NA	NA	NA
Reishofer et al., 2018	-	-	NA	NA	NA

Sadchatheeswaran et al., 2019	-	-	NA	NA	NA
Schmid et al., 2002	-	-	NA	NA	NA
Taniguchi & Tokeshi, 2004	✓	✓	0.23/0.24/0.09	Weak	Positive
Thomaz et al., 2008	✓	✓	0.49	Strong	Positive
Tonetto et al., 2014	-	✓	NA	NA	NA
Torres-Pulliza et al., 2020	✓	✓	0.046	Weak	Positive
Vorsatz et al., 2021	-	-	NA	NA	NA
Warfe et al., 2008	✓	✓	0.08 to 0.80	Both	Positive
Yanovski et al., 2017	-	-	NA	NA	NA
Young et al., 2017	-	-	NA	NA	NA
Zhou et al., 2017	-	-	NA	NA	NA
Zhou et al., 2018	-	-	NA	NA	NA

Appendix S2: Case study of Torres-Pulliza et al. (2020)

Here we present a critical evaluation of a study by Torres-Pulliza et al. (2020), who derived formulas relating geometric quantities of 2D surfaces, and used these to make claims about how fractal dimension and rugosity can be used to characterize such surfaces. Let S be the slope of average height range versus linear extent across two scales of a 2D surface:

$$S = \frac{\log(\Delta H/\Delta H_0)}{\log(L/L_0)} \qquad (S2.1)$$

where ΔH and ΔH_0 are the average height range at the large and small scale, respectively, and L and L_0 are the corresponding linear extents of the two scales. Rearranging Eq. (S2.1) and subtracting $\log(\sqrt{2}L_0)$ from both sides gives

$$\log\left(\frac{\Delta H_0}{\sqrt{2}L_0}\right) + S\log\left(\frac{L}{L_0}\right) = \log\left(\frac{\Delta H}{\sqrt{2}L_0}\right)$$

Now define a quantity

$$X = \sqrt{\frac{\Delta H_0^2}{(2L_0)^2} + 1}$$

which allows us to write

$$\frac{1}{2}\log(X^2 - 1) + S\log\left(\frac{L}{L_0}\right) = \log\left(\frac{\Delta H}{\sqrt{2}L_0}\right) \qquad (S2.2)$$

We emphasize that Eq. (S2.2) is mathematically guaranteed to be true for any surface, even a non-fractal surface. But Torres-Pulliza et al. (2020) make three stronger claims: (i) they claim that X is a measure of rugosity; (ii) they claim that S is related to fractal dimension via the formula $D = 3 - S$; and (iii) they claim, based on (i) and (ii), that Eq. (S2.2) therefore relates rugosity, fractal dimension and height range, and that "any one of the surface descriptors can easily be expressed in terms of the other two".

We identify two main problems with these claims. The first problem relates to claim (i) about rugosity. The quantity X is exactly equal to rugosity only in the idealized case where the highest corner of every grid cell is exactly a height ΔH_0 above the lowest corner and the two intermediate corners are $\Delta H_0/2$ above the lowest corner (Extended Data Figure 2 in Torres-Pulliza et al. (2020)). We show in Figure S2.1 that in general X exhibits deviations from true rugosity R, suggesting that Eq. (S2.2) may not be accurate if true R is used in place of X.

The second problem relates to claim (ii) about fractal dimension. The estimator $3 - S$ does in theory give the correct fractal dimension for an idealized mathematical object that is truly fractal. But, as explained in the main text, measuring fractal dimension from a digital surface is no trivial matter. Even if the underlying physical object is close to fractal—itself a big assumption—methods to estimate fractal dimension are subject to a range of biases, which are stronger at small and large scales. Because Eq. (S2.1) estimates S from information only at the smallest and largest scales, the estimator $3 - S$ is likely to be a very poor estimator of fractal dimension in practice.

Thus we see that, although Eq. (S2.2) does give the exact mathematical relationship between the quantities X, S and ΔH_0, claims that it continues to work well we assume $R \approx X$ and $D \approx 3 - S$ are questionable. To investigate this further, we rearranged Eq. (S2.2) to give

$$\Delta H = \sqrt{2} L_0 \left(\frac{L}{L_0}\right)^S \sqrt{X^2 - 1} \qquad (S2.2)$$

This formula holds exactly, as one can easily confirm with simulated fractal surfaces (Figure S2.2a). Now consider the corresponding approximate formula

$$\Delta H \approx \sqrt{2} L_0 \left(\frac{L}{L_0}\right)^{3-D} \sqrt{X^2 - 1} \qquad (S2.3)$$

The accuracy of this formula compared to the exact Eq. (S2.2) is substantially diminished, as shown by application to simulated fractal surfaces where true D is known (Figure S2.2c). If we instead measure D via box-counting at intermediate scales (which tends to yield reliable results; see main text, Figure 4), the formula is similarly inaccurate (Figure S2.2d). In fact, formula (S2.2) is accurate only if we use an inaccurate estimator for fractal dimension, e.g., the variation method across all scales (Figure S2.2b). Again, this undermines the claim of Torres-Pulliza et al. (2020) that Eq. (S2.2) can be interpreted as a relationship between rugosity, fractal dimension, and height range.

But how do we reconcile this with the finding of Torres-Pulliza et al. (2020), shown in their Figure 2d, that measured R, D and ΔH values fall almost perfectly on a 2D plane, suggesting that their formula is indeed accurate and that "any one of the surface descriptors can easily be expressed in terms of the other two"? The answer is simply that they did not actually test whether R, D and ΔH fall on a plane, but instead whether X, S and ΔH fall on a plane, which they are mathematically guaranteed to do. The only error that comes into their analysis arises from their measurement of S from a linear regression of height range on linear extent across multiple scales, rather than just two scales as in Eq. (S2.1). But this error is fairly minimal (e.g., Figure S2.2d) and in their case leads to $R^2 = 0.98$ instead of the guaranteed $R^2 = 1.0$ they would have obtained if Eq. (S2.1) had been used directly.

We have gone through this case study not to single out Torres-Pulliza et al. (2020), but to demonstrate that confusion surrounding geometric metrics of complexity in ecology is pervasive and that interpretations about how metrics relate to one another are fraught with difficulties. Thus, one should generally be wary of sweeping claims, such as that "three structure descriptors" can explain "98% of surface variation" (abstract of Torres-Pulliza et al.

2020), which in this case we have revealed to be closer to a mathematical truism than a grand statement about nature.

A coda to all this is that Torres-Pulliza et al. (2020) themselves make various contradictory claims. For instance, they claim at one point that "the three descriptors explain more than 98% of the variation in fractal dimension D", which does not make sense because D is itself one of the descriptors. And they claim that "All three descriptors are essential for capturing structural complexity because they explain different elements of surface geometry", despite their main claim that the three descriptors lie on a plane and thus that one of them is redundant. It is perhaps not worth reading too much into these statements, but they do reinforce the notion that confusion reigns when it comes to measuring complexity in ecology.

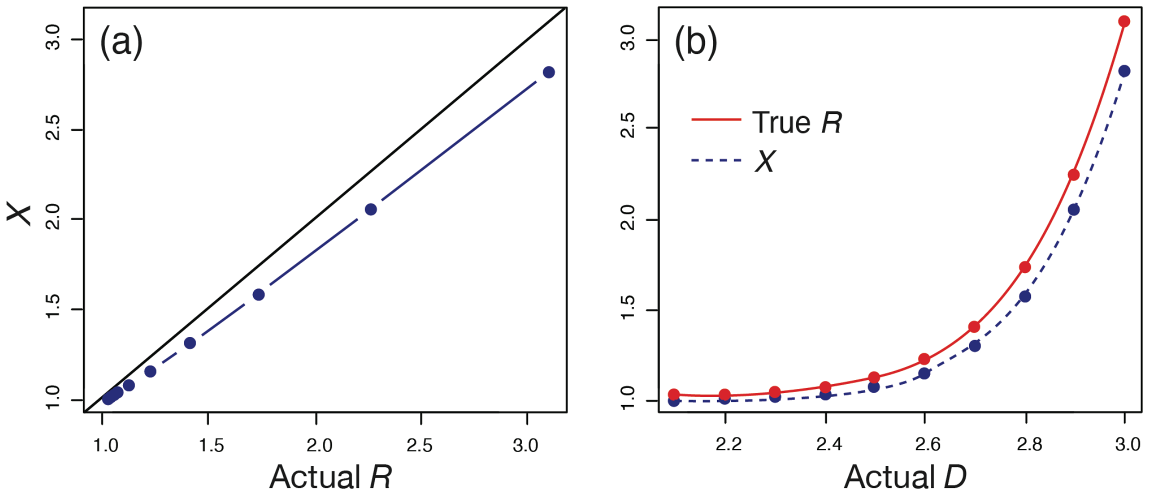

Figure S2.1 The rugosity metric X of Torres-Pulliza et al. (2020) exhibits deviations from true rugosity R. Results are generated using two-dimensional binary maps of dimension 4097 × 4097 from a midpoint-displacement algorithm with values of H ranging from 0.01 to 0.99 (see example in Figure 2a). **(a)** Plot of the rugosity metric X against R measured directly as the summed surface areas of all triangles comprising a triangular mesh generated from each map; **(b)** Plot of X and R versus fractal dimension D.

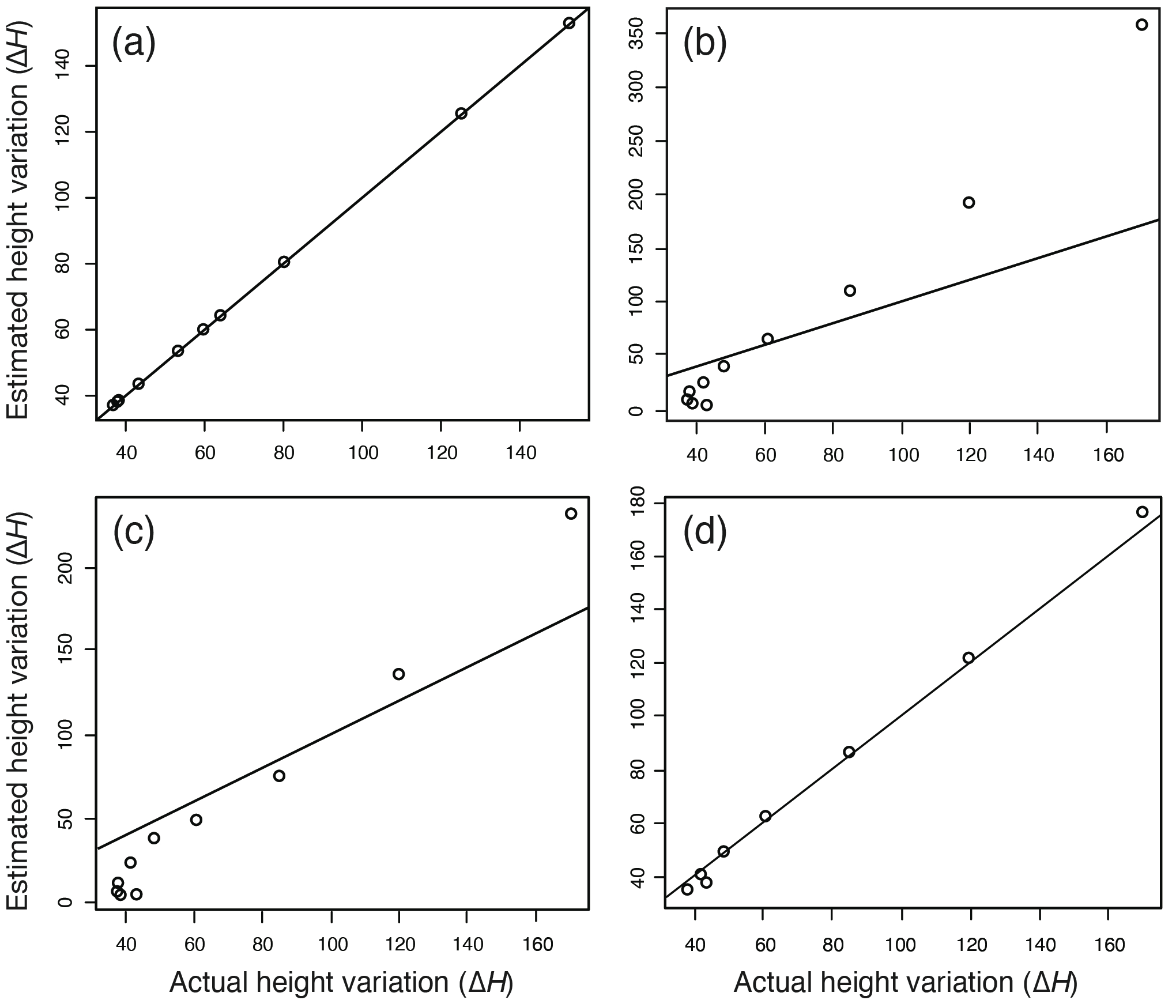

Figure S2.2 The framework of Torres-Pulliza et al. (2020) gives accurate estimates of height variation in a 2D surface only if fractal dimension D is measured inaccurately. Results are shown for **(a)** the exact formula, Eq. (S2.2), which instead of D uses S, the log–log slope of height range versus linear extent across two scales; **(b)** the approximate formula, Eq. (S2.3), which replaces S with $3 - D$, where D is the fractal dimension, and where here we have used the known fractal dimensions that were used to generate the maps; **(c)** the approximate formula but with D now estimated using the box-counting at intermediate scales, which is a reliable method (Figure 4 in main text); and **(d)** the approximate formula, but with D now estimated using the variation method across all scales, which is an unreliable method that gives inaccurate estimates of D (Figure 4 in main text).

Appendix S3: Supplementary information for Box 2

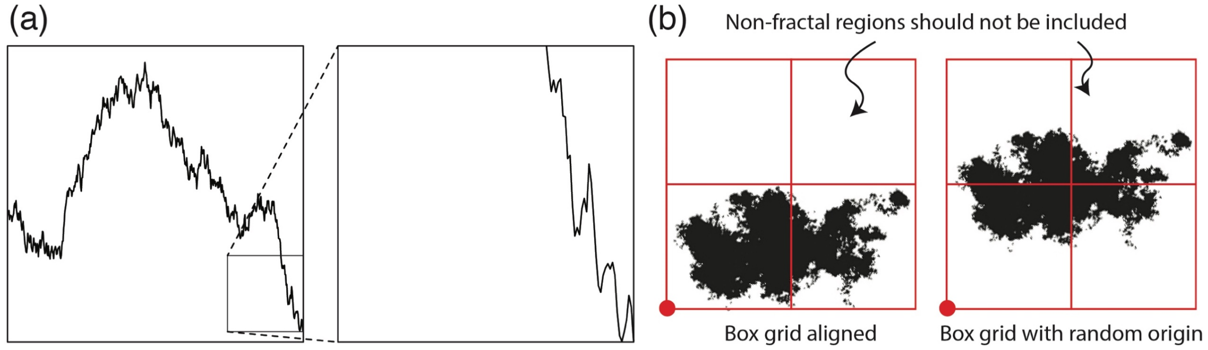

Figure S3.1 (a) Example of a 1D fractional Brownian motion (fBm) with $H = 0.5$. (b) Hypothetical example of how estimations of D using box counting can be different depending on where the box grid is placed.

Assumptions of regression methods used to estimate fractal dimension *D*

The four assumptions of standard linear regression methods are (a) linearity of the true relationship; (b) homoskedasticity of residuals; (c) independence of observations; and (d) normality of residuals. When testing whether an object is truly fractal, all of these assumptions are potentially violated.

For (a), (b) and (d), residual plots should be inspected. If substantial violations of the assumptions are observed, alternative regression methods that do not make such strong assumptions should be used. If there is substantial non-linearity in the residuals, this suggests that Eq. (B1.1) is not a good model and that the object may not be fractal; alternative non-linear models can be fit to see if the object is multifractal. Assumption (c) is always violated in box-counting because the box counts at different scales are not independent. Several methods are available to mitigate this (Reeve, 1992; Da Silva et al., 2006) but apparently not widely used.

When measuring D for an object that is assumed to be fractal, assumption (a) is true by definition, and violations of assumption (b) are less important because they affect statistical significance tests but do not bias coefficient estimates. Violations of assumption (c) can in general bias the coefficient estimates in linear regression, but we do not know of any study that has quantified this for applications to box counting specifically. Violation of assumption (d) is likely to be common because there is no theoretical reason why the distribution of $\log N(\epsilon)$ for given ϵ should be normal.

Appendix S4: Supplementary results of empirical investigation in Figure 4

In Figure 4 in the main text, we tested standard methods for estimating fractal dimension by applying them to simulated maps generated by a midpoint-displacement algorithm. Here we present the results of a similar analysis using simulated maps generated instead by a Gaussian random field algorithm, but using only the box-counting algorithm to estimate fractal dimension. Although errors were overall somewhat larger than in Figure 4, the results were qualitatively similar, with the box-counting algorithm being most accurate for intermediate box sizes $\epsilon \approx 64$ (Figure S4.1).

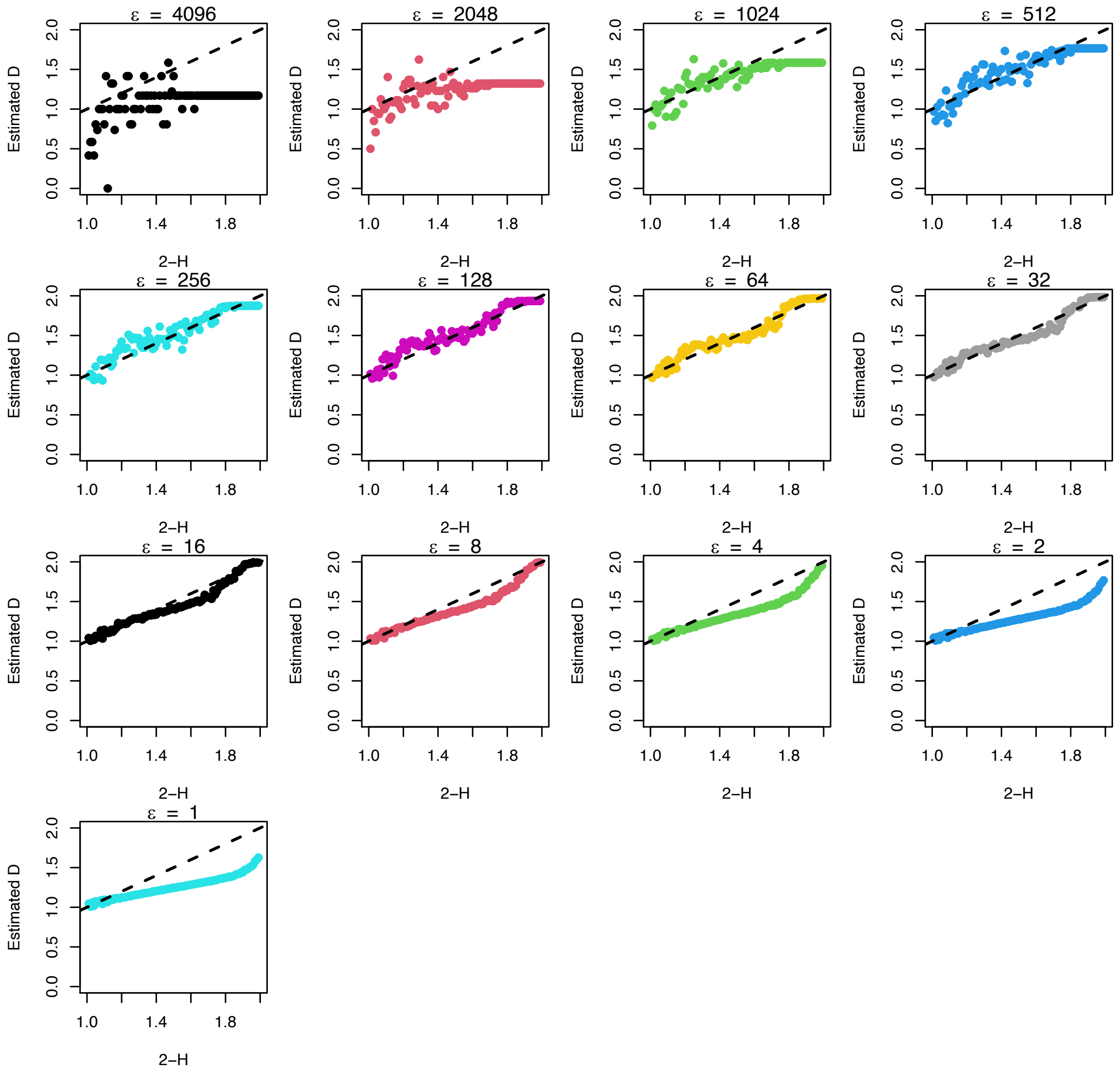

Figure S4.1 Estimated fractal dimension using the box-counting algorithm (D; vertical axes), versus true fractal dimension ($2 - H$; horizontal axes), for simulated fractal maps generated by a Gaussian random field algorithm. Panels correspond to different box sizes (ϵ) in the box-counting algorithm.

Appendix S5: Empirical demonstration of the limitations of D

To demonstrate how issues with the measurement of D are not just a minor technical inconvenience without practical significance, and that estimation errors can easily lead to different interpretations of real-world data, we conducted a small field study to compare the relationship between complexity (as measured by D) and species richness (S) in two different habitats: natural rocky shores (RS) and artificial rip-rap seawalls (SW). As expected, different values of D were obtained depending on the resolution at which D is measured, leading to different relationships between D and S (Fig. S5.1) which makes any interpretations of these results tenuous.

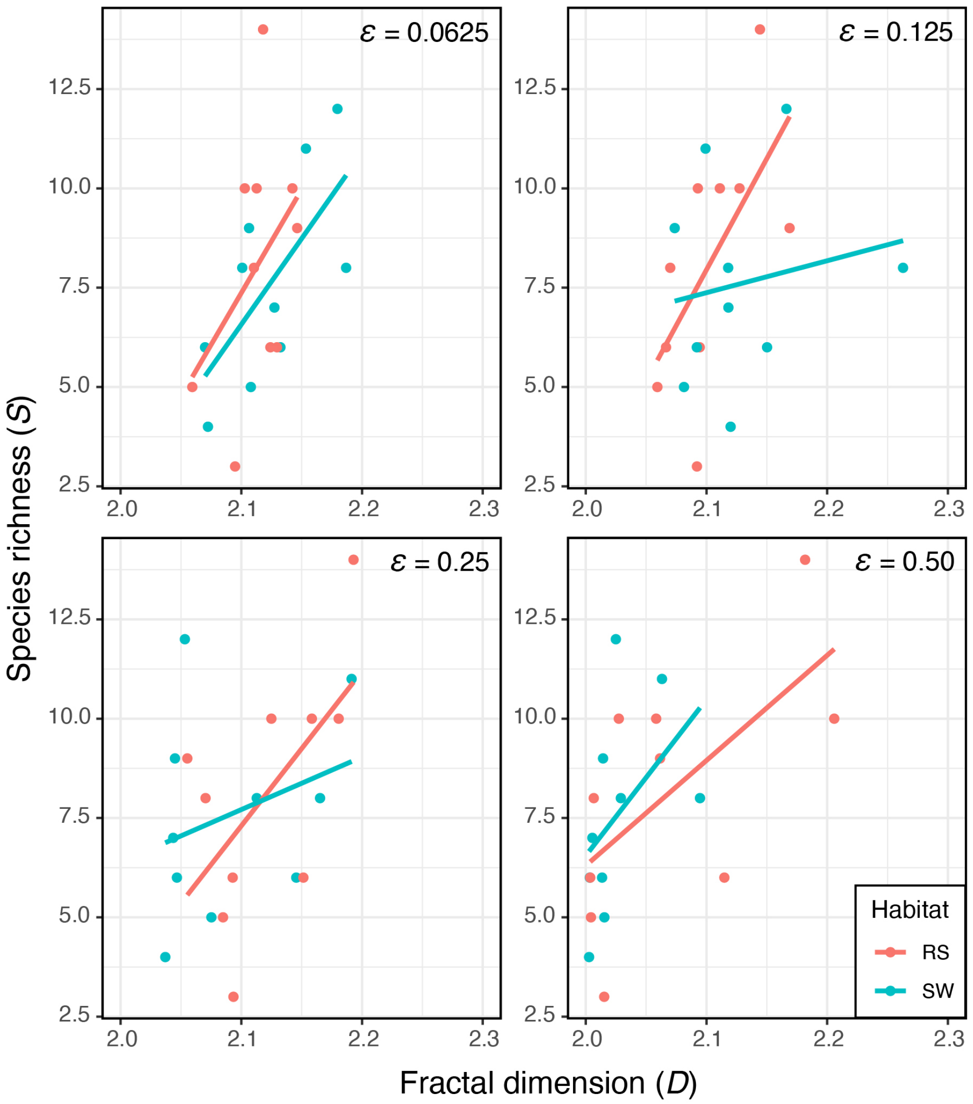

Figure S5.1 Illustration of how different relationships between species richness (S) and fractal dimension (D) can be obtained depending on the resolution at which D is measured. Here, we conducted a field study to compare two different habitat types: natural rocky shores (RS) and artificial rip-rap seawalls (SW). Ten 1 m x 1 m scans of each habitat were performed to obtain DEMs (via close range photogrammetry) from which we estimated the fractal dimension D at four different resolutions ($\varepsilon = 0.0625, 0.125, 0.25, 0.50$). The fundamental problem is that different values of D are obtained at each resolution, due to a combination of sample size limitations (number of pixels) and the fact that the underlying object is likely multifractal, rather than truly fractal as uncritical measurements of D assume (see Section 2.1.2 in main text).

References

Aria, M. & Cuccurullo, C. (2017) bibliometrix: An R-tool for comprehensive science mapping analysis. *Journal of Informetrics*, 11, 959–975.

Da Silva, D., Boudon, F., Godin, C., Puech, O., Smith, C. & Sinoquet, H. (2006) A critical appraisal of the box counting method to assess the fractal dimension of tree crowns. *Lecture Notes in Computer Science*, 4291, 751–760. doi: 10.1007/11919476_75

Reeve, R. (1992) A warning about standard errors when estimating the fractal dimension. *Computers & Geosciences*, 18, 89–91.

Torres-Pulliza, D., Dornelas, M. A., Pizarro, O., Bewley, M., Blowes, S. A., Boutros, N., et al. (2020) A geometric basis for surface habitat complexity and biodiversity. *Nature Ecology & Evolution*, 4, 1495–1501.
